# Sustainable Filtering Systems to Reduce Microfiber Emissions from Textiles during Household Laundering

**DOI:** 10.3390/polym15143023

**Published:** 2023-07-12

**Authors:** Francisco Belzagui, Carmen Gutiérrez-Bouzán, Fernando Carrillo-Navarrete, Víctor López-Grimau

**Affiliations:** Institute of Textile Research and Industrial Cooperation of Terrassa (INTEXTER), Universitat Politècnica de Catalunya—Barcelona Tech, Colom 15, 08222 Terrassa, Spain

**Keywords:** microplastic, microfiber, filtering device, textile washing, LDPE

## Abstract

During laundering, synthetic textiles (polyester, polyamide, etc.) can release small fiber debris with a length of <5 mm. These are a type of microplastics (MPs), usually referred to as microfibers (MFs), which are considered high-concern pollutants due to their continuous and cumulative entrance into the environment. Currently, as far as we know, there are no feasible alternatives to remove them. In this work, four new and sustainable filtering systems are proposed to retain the MFs emitted from domestic washing machines. The filters contain a replaceable cartridge partially filled with recycled low-density polyethylene pellets. The four designed filtering systems of different sizes were tested in a household washing machine determining the retention efficiency of the MFs after several washing cycles. It was found that all four assessed filter arrangements have a good performance for retaining MFs from the washers’ effluents. Filter F1 (diameter of 4 cm and a height of 30 cm) started retaining more than 50% of the MFs, at the 10th washing cycle, the retention climbed to 66%, while in the 20th washing cycle, its retention was greater than 80%. MFs retention was higher for filter F2 (diameter of 6.3 cm and a height of 41 cm), achieving a performance greater than 90% in the 20th washing cycle. Filter F3 was arranged by turning the F1 model flow upside down and the retention efficiency is higher compared with filter F1 values, reaching a retention efficiency of almost 100% in the 15th washing cycle. Finally, filter F4 arrangement was developed using the existing washing machine filter, obtaining better performance than the F1 and F2 filters, reaching efficiencies higher than 90% at the 20th washing cycle. In summary, depending on the arrangement, the microfiber retention efficiency was estimated between 52% and 86% in the 1st washing cycle and up to 83% to 99% in the 20th. Additionally, all arrangements demonstrated that the cartridges may last for more than 30 washing cycles before needing to be replaced.

## 1. Introduction

A significant problem facing society in the 21st century is to find routes for effective waste management. In developed countries, almost all of the plastic waste generated is recycled, burned, or disposed in landfills correctly. Only when plastic is improperly managed does it enter rivers and oceans. So, to solve the ocean plastic problem and to avoid environmental damage, strategies like encouraging the development of efficient alternatives for petroleum-based plastics, promoting reuse and recycling, and legislation to restrict the negative effects of the use of plastics are crucial, especially in developing countries where plastic pollution of the world’s oceans occurs most frequently [1,2].

According to their size, sources of plastic pollution in the ocean can be divided into macroplastics and microplastics (MPs), with the latter posing serious harm to aquatic life since they can cause bioaccumulation. Following the definition of the European Chemical Agency (ECHA), microplastics (MPs) are fragments of chemically modified and/or non-biodegradable polymers with a length of <5 mm [3]. These particles have been widely encountered polluting every assessed ecosystem [4]. The estimations of the MPs concentration in the oceans are between 15 and 51 trillion buoyant MPs in the oceans and 14 million tons in the top 9 cm of sediments of the oceans [5,6,7]. Primary MPs are those emitted into the environment in a MPs size range (length between 100 µm and 5 mm); whilst secondary are those generated in the environment from larger plastic debris [8]. On the one hand, the most prevalent sources of primary microplastics comprise plastic pellets, synthetic textiles, tires, road markings, marine coatings, and personal care products. On the second hand, secondary microplastics include microplastics coming from the fragmentation of bags, bottles, and ropes [9]. Regarding their impacts, one of their more significant is their ingestion across the trophic chain [10,11,12,13]. Additionally, some effects of the MPs on organisms have been found, for instance, their retention and endocrine disruption, among others [14,15,16,17]. These particles can behave as vectors for organisms and hydrophobic toxic compounds [18,19]. This contamination has been also found in products for human consumption and polluting the air [20,21,22,23,24,25,26], hence, there are many pathways for human exposure to MPs [27]. Nevertheless, the potential risks for human health are still unknown [28,29,30].

The microfibers (MFs) are one type of MPs which have a length to diameter ratio of >3 and a maximum length of 15 mm [3]. Textile MFs are among the most renowned as these have been widely found in the environment. These can be generated in the manufacturing, use, cleaning, and final disposal of a textile article [8]. This study is focused on those generated in the household laundering process, which can detach millions of MFs per washing cycle. The shed MFs can end up entering the aquatic environment since the wastewater treatment plants, when there are any, cannot capture one hundred percent of the MFs [31,32]. Consequently, until more ambitious pollution prevention measures are implemented, some solutions have been proposed to avoid contamination by this route. For instance, in-drum accessories to reduce the generation of MFs or out-drum filters to retain the already generated ones [33,34,35]. However, in the “retaining” alternatives, none of the existing technologies has a final treatment for the MFs.

This article aims to evaluate the performance of a new MFs filtering technology. This system can be applied to retain MFs in the equipment where these are emitted, like washing machines and dryers, among others. The principal novelty is that this system uses recycled thermoplastic pellets (low-density polyethylene (LDPE)) as the filtering media. Different arrangements of the technology were tested to know which one is more efficient for the purpose of catching MFs. Finally, the outcomes of this filter were compared with results reported in papers that tested other devices used with the same purpose. One of the main advantages of this filter is that once exhausted, the retained MFs can be immobilized. The filtering media, consisting of thermoplastic pellets, can be merged, providing the MFs with a matrix where these will be entrapped forming different types of composites.

In summary, the aim of this work is to evaluate the performance of four new filtration devices that use recycled LDPE pellets against the retention of MFs released during domestic washing. MFs retention efficiency has been experimentally determined comparing the performance of the four proposed devices. In addition, the efficiency of the studied filter arrangements was compared with alternative proposals that have been previously published by other authors.

## 2. Materials and Methods

### 2.1. Tested Filtering Arrangements

**Figure 1:**
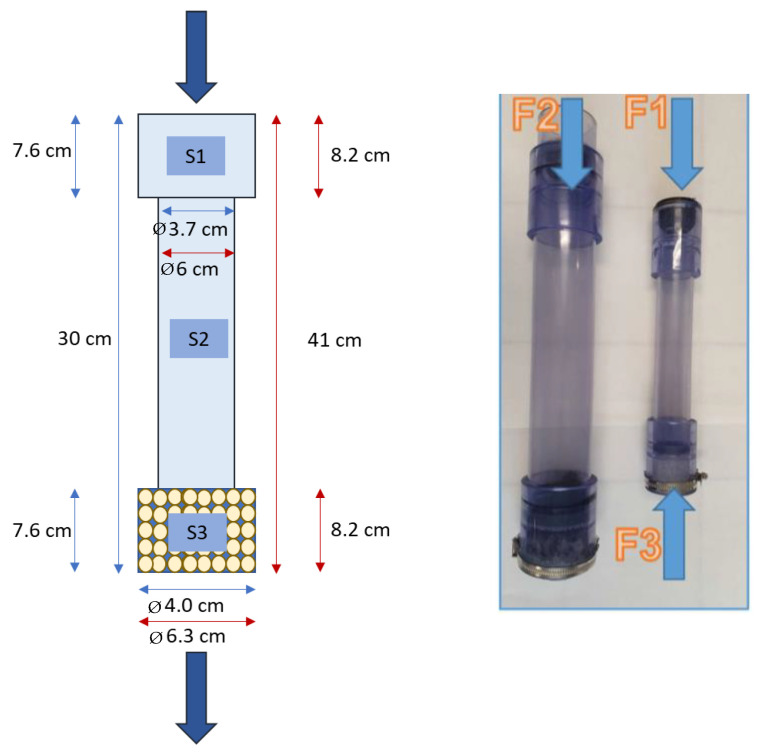
Scheme for the F1, F2, and F3 arrangements. The blue arrows show dimensions for F1 and F3. The red arrows show dimensions for F2. The external filters (F1, F2, and F3) were made of translucent PVC to be able to observe their inside while conducting the experiments and they were connected to the water outlet pipe of the washing machine.

**Figure 2:**
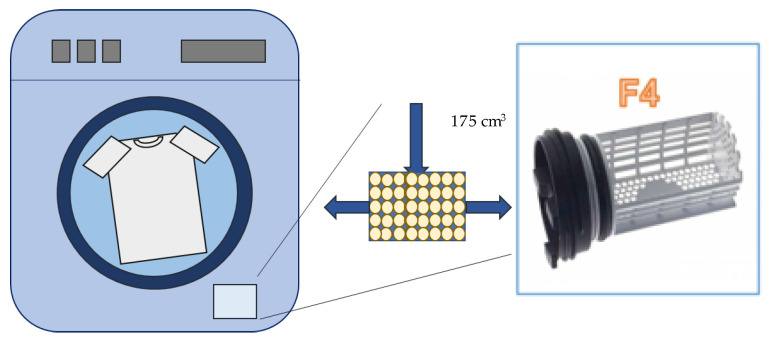
Scheme for the F4 arrangement corresponding to the existing washer filter having 5 cm of diameter and a total height of 9 cm. Blue arrows indicate the flow direction.

Four different filtering arrangements were tested in this study. Three of them were external filters, named as F1 (filter 1), F2 (filter 2), and F3 (filter 3). The fourth arrangement was a filter cartridge designed to be placed inside the existing washing machine filter (filter 4, F4). Schemes and dimensions of the arrangements are shown in Figure 1 (F1, F2, and F3 filters) and Figure 2 (filter F4). It should be noticed that the filter arrangement F3 is the same as the F1 arrangement but changing the flow direction, that is, the effluent flowed from the bottom to the top, with the filter cartridge at the top. The filters basically have three main sections: (S1) a coupling sub-system to the washing machine, (S2) an empty section to facilitate the water flow and to provide space for the accumulation of MFs and dirt, and (S3) the filter cartridge filled with pellets; whereas the F4 arrangement leverages the internal filter of the washing machine to place the cartridge inside. The novelty of these systems is the application of recycled thermoplastic polymers as the filtering media. In this work, recycled LDPE was employed in the form of pellets. These were usual commercial pellets, with a size of 3 to 5 mm on the bigger axis, and of 2 to 3 mm on the smaller axis. All the LDPE pellet cartridges were structured to have a density of 0.5 to 0.6 g/cm^3^.

PVC was selected as the filter structure material due to its higher resistance to aging compared to other polymers commonly used in water pipes, such as HDPE or PP. In this sense, Zhang et al. [36] confirm in aging tests of rainwater facilities that PVC releases a quantity of MPs almost 12 times lower than HDPE. On another hand, regarding the material contained in the replaceable cartridge, LDPE was selected because of its high affinity for the retention of MPs, as can be seen in previous literature where an LDPE film is used to capture and sample MPs released from PVC pipes [37].

### 2.2. Materials and Pre-Treatments

Two types of black commercial polyester fabrics were selected. One was a woven fabric while the other was a fleece-knitted fabric. For each experimentation, an equal number of pieces and weight (20 pieces, 280 g each piece) were distributed in two identical commercial washing machines (FAGOR Innovation F-2180, FAGOR, Mondragón, Basque Country, Spain), one for the control and the other to test each of the filtering arrangements. In this way, 70% (5.6 kg) of the maximum weight of these washers (8 kg) was introduced in each washing machine.

Before data compilation, two independent pre-treatments were made. On one hand, the washing machines were cleaned by doing two empty washing cycles. On the other hand, the fabrics were washed for five consecutive washing cycles prior to data collection. The latter pre-treatment was performed to achieve a constant detachment rate of MFs, which has been reported to be from the 5th washing cycle [31,38].

Afterward, the MFs filtering arrangements were independently tested versus a normal discharge of a washing cycle. The filtering arrangements were connected to one of the washers, while the other washer was kept unmodified to obtain comparable reference data of the concentration of MFs in the effluent. Then, the fabrics were washed with the “cold” program (30 min, 22 L of effluent, 1000 RPM, water grid temperature ~25 °C). A common detergent (Bosque Verde, SPB, Cheste, Valencia, Spain) was introduced to the washing trials with a volume of 75 mL per cycle.

The effluent of the washing cycles 1, 5, 10, 15, and 20 were collected and evaluated by gravimetry. For this, from each effluent, a sample of 10 L was separated while continuously stirring. Then, from this 10 L sample, 2 aliquots of 2 L were filtered through 20 µm polyamide filters. The polyamide filters were dried and weighed before and after the filtration of the discharged water. The difference between the two weights of the filter was considered as the detached MFs. As each filter weight has 0.1 mg uncertainty and the filter is weighted twice (before and after filtering), we consider that the values of the MFs have an uncertainty of ±0.2 mg. The retention efficiency of the filtering arrangements was calculated as the relation between the MFs found in the washing effluent with the filter device versus the washing effluents obtained without the filtering system (See Equation (1)). The results were expressed in the percentage of retained MFs:

(1) $$R=\left[1-\frac{\left(D_{2}-D_{1}\right)}{\left(N_{2}-N_{1}\right)}\right]\cdot 100\%$$

where *R* is the retention efficiency, %;

$D_{2}-D_{1}$ is the difference between the mean values of the weight of the filters when a filtering arrangement was applied, mg, and
$N_{2}-N_{1}$ is the difference between the mean values of the weight of the filters when no filtering arrangement was applied, mg.

In order to find the replacement time intervals of the cartridges, after the 20th washing cycle, the filtering arrangements were operated until they were clogged. In these trials, the efficiency was not measured.

### 2.3. Permeability Coefficient and Porosity

The permeability coefficient was evaluated by applying Darcy’s Law. The purpose was to determine the resistance to the flow of the devices when using LDPE as the filtering media with the conditions explained in the Filtering Arrangements section, specifically, the density of the pellets. Darcy’s Law can be expressed by Equation (2):

(2) $$Q=K\frac{\Delta h}{L}A$$

where Q is the flow of the fluid, cm^3^/s; K is the permeability coefficient, cm/s; Δh is the difference between the heights of the fluid at the influent and effluent of the filter (cm); L is the height of the filtering section, cm, and A is the area of the filtering section, cm^2^.

On the other hand, the porosity was calculated by introducing LDPE pellets with a density of 0.5 to 0.6 g/cm^3^ in a 100 mL recipient. Then, water was poured into the recipient and its volume was measured. The porosity was calculated with Equation (3):

(3) $$\emptyset =\frac{V_{W}}{V_{T}}$$

where ∅ is the porosity (dimensionless) and $V_{W}$ is the volume of water poured into the $V_{T}$ 100 mL recipient filled with pellets.

## 3. Results and Discussion

### 3.1. Efficiency of the Filtering Arrangements

**Figure 3:**
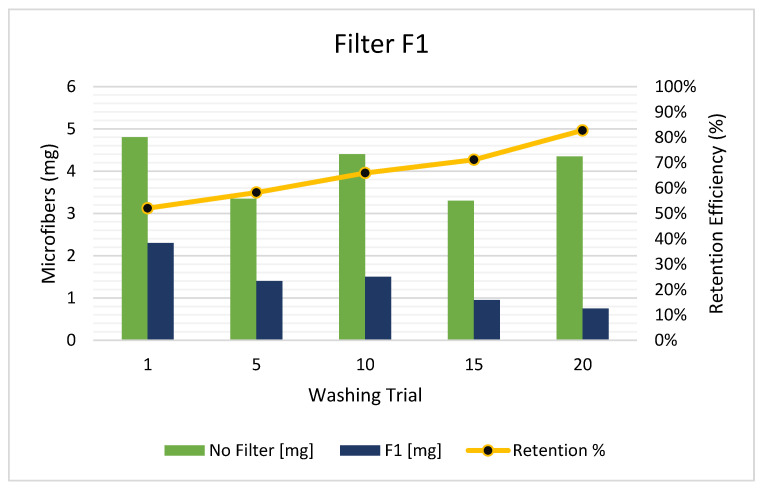
Released MFs in the effluent with F1 arrangement (outside smaller filter).

The four filtering arrangements described in the methodology section have been tested to evaluate their MFs retention performance. All the arrangements have shown a statistically significant difference between using or not using the filtering system (*p* < 0.05 for all the cases, ANOVA). Regarding the tested models, as can be seen in Figure 3, the F1 arrangement has started retaining more than 50% of the MFs. It has also shown a constant and positive efficiency growth. Hence, at the 10th washing cycle, the retention reached 66%, while in the 20th washing cycle, its retention was greater than 80%. In Figure 3, it can also be seen that in the last tested washing cycles, the mass of MFs found in the filtered effluent was lower than 1 mg.

**Figure 4:**
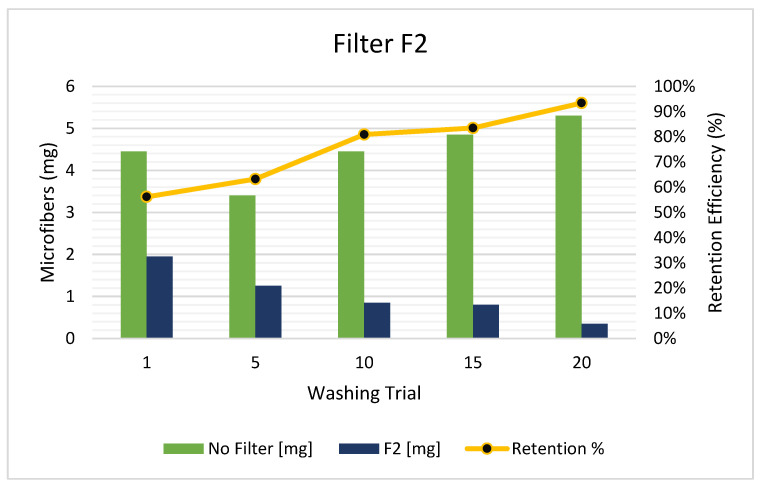
Released MFs in the effluent with F2 arrangement (outside larger filter).

Additionally, the F1 arrangement was found to have a cartridge replacement time interval of approximately 30 washing cycles before clogging. On the other hand, the F2 arrangement showed a similar trend. As explained before, the F2 was larger and had more length and volume of filtering media (LDPE pellets) than the F1. The F2 filtering model has shown a greater MFs retention performance since the beginning (see Figure 4). In this line, the F2 model started with an MFs retention greater than 55%; in the 10th washing cycle, it was already greater than 80%, reaching the performance that the F1 arrangement had at the 20th cycle. The F2 filter achieved a performance greater than 90% in the 20th washing cycle. This can be explained as a consequence of the larger length and volume of section S3, which gives more flow resistance and retention time to the MFs. Also, the volume of section S2 was greater to give space and time for the filtering process to occur, meaning that the filtering can be conducted smoothly.

As can be seen in Figure 4, in the last washing cycles, the MFs that were found in the effluent approached zero. Moreover, an extra advantage of using a larger filter is the increase in the replacement time interval of the cartridges and the subsequent comfort for the users. In this sense, the F2 arrangement was able to handle approximately more than 40 washing cycles before clogging, at least 10 more than the F1 model.

**Figure 5:**
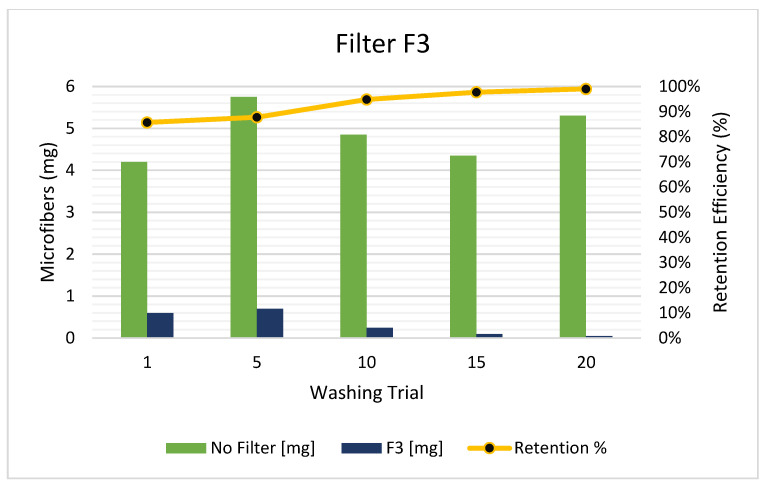
Released MFs in the effluent with F3 arrangement (outside smaller filter with the bottom-up flow).

As explained in the methodology section, the F3 filter model was arranged by turning the F1 model flow upside down, that is, the effluent flowed from bottom to top. As can be seen in Figure 5, the F3 arrangement started with a retention efficiency greater than 85%. This means that the F3 has shown an initial higher performance in contrast with the other tested arrangements. Moreover, from the 15th washing cycle, the retention efficiency was almost 100%, indicating that the mass of MFs in the effluent was almost completely eliminated. Moreover, the replacement time interval of the F3 cartridge was also improved in contrast to the F1 model reaching approximately 40 washing cycles. As these were identical in size, the conceived reason for the improvement was that the MFs did not create a blockage at the begging of the filtering section (S3). Hence, the MFs were easily retained in the previous section (S2) and the filtering process was smoother as well.

**Figure 6:**
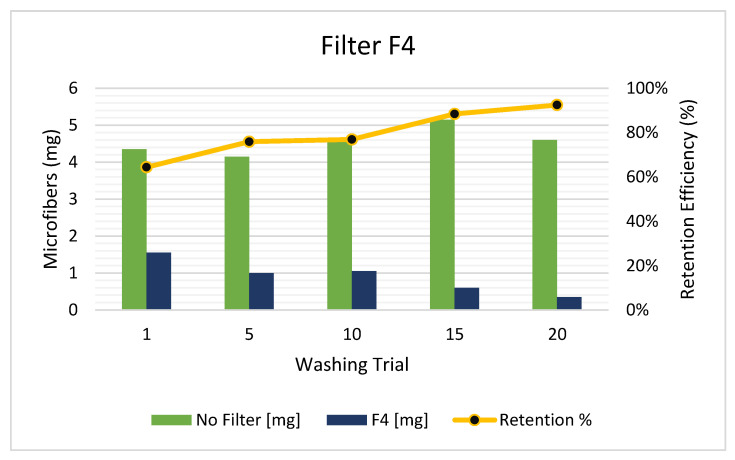
Released MFs in the effluent with F4 arrangement system introduced in the existing washer filter.

Finally, as mentioned before, the F4 arrangement was developed to be installed using the existing washing machine filter. This arrangement has shown a better starting performance than the F1 and F2 filters (see Figure 6). This might be explained as a consequence of the roughly radial filtering process that it ineluctably has (it has a concentric flow from the inside to the outside). The F4 started with 65% of efficiency and reached >90% at the 20th washing cycle. As the F4 is constructed by using and surrounding the existing washer filter, the cartridge lifetime will depend on its size. Yet, in washers with small, designed filters, a prolongation of the existing filter can be designed. In this way, the main advantage of this model is that it does not need the installation of external arrangements. Hence, the washers that have no space for external filters or that have the effluent hose welded to the discharge tube can still have an alternative to install a catcher device for MFs. It should be mentioned that all the arrangements surpass the minimum expected by the users as an “optimal” replacement frequency (found at 17 washes by [39]).

**Figure 7:**
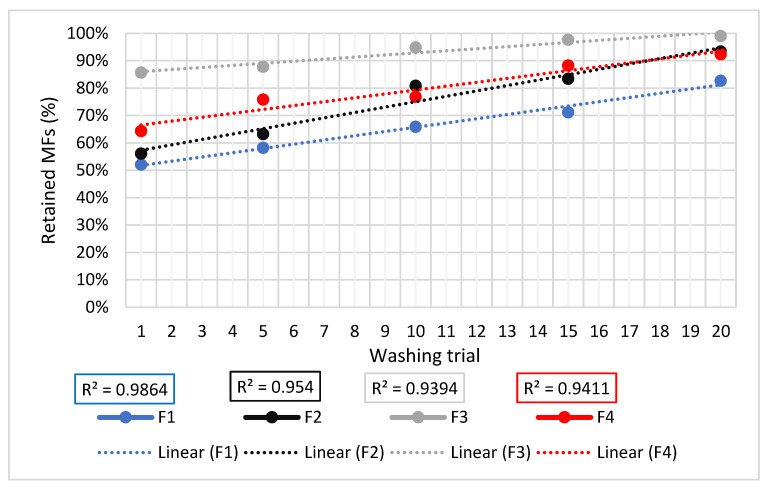
Comparison of MFs retention efficiency for all the filtering arrangements.

Theoretically, in all the cases, the filtering capacity of this system is determined by two main aspects. First, the lint that is formed between the MFs on and through the LDPE pellets. This latter feature provides the filter with an initial bump to start. In other words, the already retained MFs strengthen the filtering capacity by forming a new lint layer that catches more MFs. This can be seen in Figure 7, where the efficiencies of the filtering arrangements were found to be variable and positive (a linear trend with R^2^ > 0.9) throughout the trials. This was also empirically seen through the translucent PVC filters, where the lint formed by the MFs was easily observed. In this sense, it was also appreciated that the lint formed “hot spots” where the accumulation was greater.

Concerning the permeability coefficient, it was estimated in the order of 10^−3^ m/s (1.3 to 1.5 cm/s). This parameter is related to the structure of the filtering media and determines the resistance to the flow that the device has when using 0.5 to 0.6 g/cm^3^ of LDPE as the filtering media. As it can be seen, the resulting value can be considered as “very high” or “rapid” when compared to other similar filtering media concerning the filtration mode (e.g., sand) [40]. In this sense, the effluent of the washers can flow through the filters without any inconvenience. This latter feature was confirmed in the experiments made to assess the filter performance. Regarding the porosity, it was found at approximately 0.35, which provides sufficient empty voids to let the water flow but also enables retaining the MFs. It should be mentioned that the filter is a dynamic system, meaning that it will change most of the main parameters over functional time. However, as was seen throughout the experiments, these alterations affect the system by increasing the filtering efficiency.

Finally, once the filters are exhausted, the thermoplastic pellets and the MFs can be reused by enclosing the MFs in the LDPE polymeric matrix by obtaining composites that can be further used for different purposes. In this regard, composites were obtained with exhausted filters as has been previously published by the authors of [41].

### 3.2. Comparison with Other MFs Filtering Devices

With respect to other commercial filters, F1, F2, F3, and F4 have presented higher performance than most of the devices found in the market (see Table 1). Particularly, the F3 arrangement has shown the best retention efficiency. Two published studies have evaluated the MFs retention efficiency of the already commercialized devices. McIlwraith et al. (2019) [42] tested the *Cora Ball* and the *Lint LUV-R*. They used 545 g of a 100% polyester fleece blanket, washing at cold temperature, 30 min total washing time, 26.5 L of effluent, and a spin speed of 660 RPM. Excluding the mass of textiles used, the conditions are similar to the ones used in this study. However, they only evaluated the MFs retention efficiency at the 1st washing cycle. The filtering procedure to assess the MFs retention was conducted through a 10 µm pore size filter. They reported that these technologies can achieve a drop in MFs from 26% (*Cora Ball*) to 87% (*Lint LUV-R*).

On the other hand, Napper et al. (2020) [43] also tested some technologies. They used three synthetic fabrics (jumpers) made of 100% polyester, 100% acrylic, and 60% polyester/40% cotton. Each washing cycle was filled with 1300 g of textiles. To avoid the initial peak that new garments have on the detachment of MFs, they also washed the clothes 4 times before data collection. The front-loading washers were set at 30 °C, 1000 RPM, and 45 min. The reported outcomes corresponded to the average of the data collected after the 1st, 5th, and 10th wash. The filter pore size used was of 1 µm. They have reported that the external device *XFiltra* [44] was the best device with an efficiency of 78%. According to their study, two key features can explain the higher efficiency of this filter: the finest diameter pore in contrast with other external devices (60 µm vs. > 175 µm), and the usage of an integrated pump, which might need extra energy supply. On the other hand, *PlanetCare* [45] affirms that their device has an MFs retention efficiency of >90%, which contradicts the findings of Napper et al. (2020) [43]. The results of these studies and ours are summarized in Table 1.

As can be seen in Table 1, different outcomes were reported for the same devices; hence, further interlaboratory trials are required to achieve definitive conclusions. In addition, it should be noted that, for a more accurate comparison, the results of McIlwraith et al. (2019) [42] must be compared with our 1st washing cycles’ outcomes, whilst the outcomes of Napper et al. (2020) [43] must be compared with the average of the outcomes obtained from the 1st, 5th, and 10th washing cycles. These averages are 59% for F1, 67% for F2, 90% for F3, and 72% for F4.

Moreover, it is important to underline that the final disposal of the retained MFs is a crucial concern still to be addressed. Napper et al. (2020) [43] indicated, for example, that once the filters are cleaned by collecting the MFs, these can be “*thrown into the everyday household waste*”. Hence, the MFs could also finish in the environment. In this work, the filtering devices are made of recycled materials and they have the additional advantage that the MFs can be further treated with the filtering media, for example, to prepare composite materials [41].

Finally, another parameter that must be considered is the time intervals to clean or replace the filters. The *Lint LUV-R* needs to be cleaned every two to three loads of laundry according to the manufacturers. *Planet Care* can be used for 20 washing cycles. *XFiltra* can handle 30 washing loads [43]. In our case, the smaller arrangements F1 and F3 were found to hold at least 30 washing cycles, whilst the F2 arrangement can withstand at least 40 loads of washing. On the other hand, the in-drum F4 arrangement replacement interval will depend on the size of the existing washing machine filter. Regarding the commercial in-drum devices needed, they need to be cleaned once every load of laundry. According to Herweyers et al. [39], the minimum cleaning time interval is between 15 and 17 washing cycles. However, not all devices meet this requirement. In the case of the system assessed in this study, the replacement time interval for the cartridges surpasses the users’ expectations.

## 4. Conclusions

As demonstrated in this research, filters are essential in retaining microfibers while washing fabrics. By trapping the microfibers and restricting their release into the environment, filters help to reduce the impact of microfiber contamination on water effluents.

Four different models of a new textile microfiber retaining system were evaluated. It was found that all the assessed arrangements have a good performance for retaining microfibers from the washers’ effluents. Depending on the model, the microfiber retention efficiency was estimated between 52% and 86% in the 1st washing cycle and up to 83% to 99% in the 20th. The best performance was found when the flow of the washers’ effluent went from bottom to top, being the filtering media was at the top, that is, for filter F3. Moreover, all the arrangements showed a sufficient replacement time interval for the cartridges, as these were capable of handling more than 30 washing cycles. It is important to mention that one of the arrangements did not need an external device as it was applied by surrounding the existing washing machine filter.

In addition to the good performance of these filters, it should be highlighted that they hold two relevant features. First, the usage of thermoplastic recycled waste for the filtering media and the shell, which strengthens the circular economy philosophy and produces a “greener” product. Second, that the retained microfibers can be further and easily immobilized in the LDPE polymeric matrix by merging the filtering media with the microfibers inside. This latter feature can be harnessed to develop different types of products, tackling one of the main issues of the existing alternatives to reduce the microfibers, which is the subsequent treatment of these pollutants.

## Figures and Tables

**Table 1:** Results reported by two different publications regarding MFs reduction devices and comparison with the present study.

Device	McIlwraith et al., 2019 [42] ^1^		Napper et al., 2020 [43] ^2^	Our Work				
	Retention Efficiency (%)			Retention Efficiency by Weight (%)				
	By Count	By Weight	By Weight					
**In-Drum**				**Washing**	**1st**	**5th**	**10th**	**20th**
Cora Ball (No mesh)	26	5	31	F4	64	76	77	
GuppyFriend (50 µm)	-	-	54					
4th element (50 µm) ^3^	-	-	21					
**External Filters**				**Washing**	**1st**	**5th**	**10th**	**20th**
Lint LUV-R (150 µm)	87	80	29	F1	52	58	66	83
XFiltra (60 µm)	-	-	78	F2	56	63	81	93
PlanetCare (200 µm)	-	-	25	F3	86	88	95	99

^1^ Outcomes are for the MFs retention average from 1st washing cycle. ^2^ Outcomes are for the average of the 1st, 5th, and 10th washing cycles’ data. ^3^ Napper et al. [43] was used for the reference of the 4th element washing bag.

## Data Availability

Not applicable.
